# Supplementary material for: Patterns of Intron Gain and Loss in Fungi
Source: PLoS Biol. 2004 Nov 30;2(12):e422. doi: 10.1371/journal.pbio.0020422 (PMC532390; doi:10.1371/journal.pbio.0020422)
Supplement: Table S1 — Also available at http://genes.mit.edu/NielsenEtAl/. (4.3 MB ZIP). [file pbio.0020422.st001.zip › NielsenEtAl/html/43.html]

AN8672.1.NCU07443.1.MG02767.1.FG08595.1


```
 CLUSTAL W (1.82) Multiple Sequence Alignments - Introns Inserted


Sequence 1: NCU07443.1	1562 aa
Sequence 2: FG08595.1	1524 aa
Sequence 3: MG02767.1	1524 aa
Sequence 4: AN8672.1	1501 aa
Alignment Length: 1612 aa
Number Identitical Residues: 678 aa
Alignment Score (without introns) 34339


MG02767.1 	--------------------------------------MTIKSSGLKRLSLLGRAQNRGS
NCU07443.1	MTPRPADEDEGGAE------PTP--ITATQRSRWATRKLTVKSSGLKRLSLKNRNNHGNA
FG08595.1 	MTTTTSADGHLAAP------DDSQHLSATQRSRWATQRKSVNSSNNKRNSILDRMGHKKT
AN8672.1  	MASDSLHPNRLEPPPPPIQQTDSNISTPTNRQRWATHRASTAGGMRKRVSIMDRFHKRME
          	 :. .   .   . ... ..  ..  :.:. .  ::   :  ..  ** *: .*  :   

MG02767.1 	STEKKRASMGKGGESLQQSSEGDNEDQDQG----EHDDQPEGPGPRTLYFNLPLPPEKLN
NCU07443.1	VGIEKKRASGHSGKSDSTAGQSDTPPPTAGGHAGKDDADEQHSEPRTIFCGLPLADEFKD
FG08595.1 	GSNEKNPPS---DGSDPAGDDGQSPEANPD----NEDEDEEHEN-RTLYFNQPLPTELLD
AN8672.1  	NKDEKRKST------LANSAAGPSSPVSPS------------EGNRKVYVNIPLPESERD
          	   :*. .          .  . .     .               *.:: . **. .  :

MG02767.1 	DKGELVDTYPRNKIRTAKYTPLSFIPKNLWLQFHNIANIYFLFLVILA0FFPIFGSVNPG
NCU07443.1	DEGHPTQQYPRNKIRTAKYTPLSFVPKNLWFQFHNIANIFFLFVVILV0IFPIFGGVNPG
FG08595.1 	ENGAPSQTYTRNKIRTAKYTPISFVPKNLWFQFHNVANIFFLFLVILV0IFPIFGGVNPG
AN8672.1  	EDGHPLANYPRNKVRTAKYTPITFVPKNLWFQFQNIANVYFLFIIILG0FFSIFGVDNPA
          	:.*     *.***:*******::*:*****:**:*:**::***::**  :*.***  **.

MG02767.1 	LGAVPLIFIVTVTAIKDAIEDSRRTQSDIELNDSTVHRLCGWNNINVKEDNISLWRRFKK
NCU07443.1	LNAVPLIVIICVTAIKDAVEDYRRTVLDNVLNNAPVHKLHGIPNVNVEADNVSLWRRFKK
FG08595.1 	LNAVPLIFIIAVTAIKDAIEDYRRTVLDIELNNAPVHRLRNWNNVNVLEGDVSTWRQFKK
AN8672.1  	LNTVPLIVIIVVTAIKDAIEDWRRTVLDTELNNSPVYRLVDWHNVNSTEDSVSLWRRFKK
          	*.:****.*: *******:** ***  *  **::.*::* .  *:*   ..:* **:***

MG02767.1 	GNTKVFGGLWRFMETLWSK---EAR--VKRREKAAQVADTPRISIDTRRTGRP--ESVIS
NCU07443.1	ANSRFFGALWHKTEGLWKK---EDDNPILKNHTASHDEPDPRMSMESRTSR---------
FG08595.1 	ANSKFFGSIWRATQSIWSK---KAKEERAKRKAVSTEEDGPRASVETQRTRQSMRQSIAS
AN8672.1  	ACTRGTIWTYRKLRVWFSKNKNHNESAFAERRASFLTTVSPRASMDSEHGDRG-------
          	. ::     ::  .  :.*....  .   ...        ** *:::.            

MG02767.1 	PHTHRTSFISAREDIPMTPVTSPLPRAEGQQNLEVVPQQEGLASGRPS--SNFSFRGDSA
NCU07443.1	---ARRSLNVVRDEVEMTPVPSPLPHQPNEMDFPGEGSSRQVDN--------------KY
FG08595.1 	PFTGRESFMSAREDIQMTPVPSPSPQATPHARFEMP-DEQDAKR--------------AT
AN8672.1  	----------EEEAIQMTPVSSPMPDARSDWPLSSSETDQHLHPDKAARRASMAPSDISV
          	           .: : ****.** *    .  :     ..    . .:  :. :  .   

MG02767.1 	ELAHIKTDLINRTIPAEGKARFRRDKWKNLQVGDFVRIYSDDELPADIIILATSDPEGAC
NCU07443.1	ALQDIKGDLVNRNLPISGKARFAKDAWKGLVVGDFVRIYNDDEIPADIIILATSDPDGAC
FG08595.1 	ALMQMKPDVINYHHPASG-ARFQKDTWKSLNVGDFVRIYNDEELPADVIILSTSDPDGAC
AN8672.1  	GAPRKAGSVVDMSKQIVGNARFKRDYWKSLQVGDFVRLYNGDPIPADIVVLSTSDPDGAC
          	       .:::      *.*** :* **.* ******:*..: :***:::*:****:***

MG02767.1 	YIETKNLDGETNLKFRQALKCGRNMKNSRDCERAQFIVESEPPQPNLYKYNGAIKWNQPI
NCU07443.1	YVETKNLDGETNLKVRSALRCGRGMKHARDCERAQFIIESEPPQPNLYKYNGAVRWLQEL
FG08595.1 	YVETKNLDGETNLKVRQAVRCGRSLKHARDCERAEFVVESEGPQPNLYKYNGAIKWKQSV
AN8672.1  	YVETKSLDGETNLKVRQALHCGRKVRHARDCERSEFIIESEAPHPNLYAYNGAVRWDQRD
          	*:***.********.*.*::*** ::::*****::*::*** *:**** ****::* *  

MG02767.1 	EGDASGSWREMTEPITIDNTLLRGCNLRNTDWVLGVVLFTGHHTKIMMNSGITPSKRPRI
NCU07443.1	PNDEDGDPIPMSEPISIDNMLLRGCNLRNTEWALGVVVFTGHDTKIMMNAGVTPSKRARI
FG08595.1 	PGYLDDEPEDMTEPITIDNLLLRGCNLRNTEWIVGVVIYTGHDTKIMMNAGITPSKRARI
AN8672.1  	PDYPDAPRKEMVEPITINNLLLRGCSLRNTEWILGVVIFTGVETKIMLNSGETPSKRPQL
          	 .  .     * ***:*:* *****.****:* :***::** .****:*:* *****.::

MG02767.1 	ARELNYNVLYNFFILFGMCLLSAIVNGFAFGTSDNSIALFEYGSIGPTPAMNGFITFWAA
NCU07443.1	ARELNINVVYNFTILLIMCLIAAIANGVAWAKTDASSYWFEWGSIGGTSGLTGFITFWAA
FG08595.1 	AREMNFNVVCNFGILLIMCLLAAIINGVAWAKTDASLHFFDFGSIGGKPAMSGFITFWAA
AN8672.1  	AKDLNWNVIYNFILLFFMCLISGIVNGVAWASDEGSLNYFET-PYGSTPAVTGIITFWVA
          	*:::* **: ** :*: ***::.* **.*:.. : *   *:  . * ...:.*:****.*

MG02767.1 	IILFQNLVPISLFISLELVRLLQAFFIYSDVDMYYEPIDQPCIPKSWNISDDLGQIEYIF
NCU07443.1	VIVFQNLVPISLYISLEIVRTLQAYFIYSDINMYYEPIDAPCIPKSWNISDDVGQIEYIF
FG08595.1 	IILFQNLVPISLYITLEIVRTLQAIFIYNDVEMYYEPIDQPCIPKSWNISDDVGQIEYIF
AN8672.1  	LILFQNLVPISLYISLEIVRTAQAIFIHSDVFMYYDKLGISCIPKSWNISDDVGQIEYIF
          	:*:*********:*:**:**  ** **:.*: ***: :. .***********:*******

MG02767.1 	SDKTGTLTQNVMEFKKATINGQPYGEAYTEALAGLHRRMG-IDVEKEAAEARVQIAADKE
NCU07443.1	SDKTGTLTQNVMEFKKATINGQPYGEAFTEAQIGMSKRSGGGDIESEIARIKDEIEQAKA
FG08595.1 	SDKTGTLTQNVMEFKKATINGQPYGEAYTEAQAGMQKRLG-IDVEKEGERVRAEIADAKV
AN8672.1  	SDKTGTLTQNVMDFKKCTVNGVSYGEAFTEAQVGLVRREG-GDADAEAARAREKIAMDTT
          	************:***.*:** .****:***  *: :* *  * : *  . : :*   . 

MG02767.1 	KALAALRKIHDNPYLHDDDLQFIAPDFVEDLMGANGQEQQQACERFMLALALCHTVIPER
NCU07443.1	RTLHGLREIHNNPYLHDEDLTFVAPDFVEDLAGKNGPEQQKANEHFMLALALCHTVVAEK
FG08595.1 	RALAGLRNIHDNPFLHDESLTFIAPDFVSDLAGESGPDQKEANEFFMLALALCHTVMAEK
AN8672.1  	RMIKMLRQMHDNPYLRDENLTFISPDYVADMGGQSGEAQKQATEHFMLALAVCHSVITEH
          	: :  **::*:**:*:*:.* *::**:* *: * .*  *::* * ******:**:*:.*:

MG02767.1 	QPGEKATMLYKAQSPDEAALVSTARDMGFTVLSSNSDGVRLNVMGEEKYYPILNTIEFNS
NCU07443.1	QPGDSPKMIFKAQSPDEAALVATARDMGFTVLGMSDGGVNVNVMGKDMHYPVLNIIEFNS
FG08595.1 	VDGDIPQMIFKAQSPDEEALVATARDMGFTVLGSSGEGINLNVMGEDRHYQILNTIEFNS
AN8672.1  	TPGDPPQIEFRAQSPDEAALVGTARDCGFTLLGRSNDDLIVNVMGEERTYTVLNTLEFNS
          	  *: . : ::****** ***.**** ***:*. .. .: :****::  * :** :****

MG02767.1 	SRKRMSAIVRMTDGQTVLFCKGADSIIYSRLKKGEQKQLRTDTAQHLEMFAREGLRTLCI
NCU07443.1	SRKRMSAIVRMPDGKIKLFCKGADSIIYARLKRGEQKELRRETAEHLEMFAVEGLRTLCI
FG08595.1 	SRKRMSSIVRMPDGRIILFCKGADSIIYSRLKRGEQKELRKTTAEHLEMFAREGLRTLCI
AN8672.1  	TRKRMSAIVRMPDRSIRLFCKGADSIIYSRLAPGKQQELRKKTAQHLETFAREGLRTLCV
          	:*****:****.*    ***********:**  *:*::**  **:*** ** *******:

MG02767.1 	AERVLGEQEYQAWSKEYAVAAAAVENREDKMEAIADQIEQDLTLLGGTAIEDRLQDGVPQ
NCU07443.1	AEKELTEQEYYEWKKEHDVAATALENREEKLEEVADKIEQDLTLLGGTAIEDRLQDGVPD
FG08595.1 	AWKEVTEHDYRVWKKEHDAAASALEEREEKLETVAELIEQDLYLVGGTAIEDRLQDGVPD
AN8672.1  	ADRKLSEEEYRAWSKEHDIAAAALTDREEKLENVASAIEQDLMLIGGTAIEDKLQDGVPD
          	* : : *.:*  *.**:  **:*: :**:*:* :*. ***** *:*******:******:

MG02767.1 	TIAVLAEAGIKLWVLTGDKVETAINIGFSCNLLNNDMELLNLKVDEDETGLTTREQFMAK
NCU07443.1	AIELLGNAGIKLWVLTGDKVETAINIGFSCNLLNNDMDLVRLQVSEDEAGVQQEAEYLRL
FG08595.1 	TIALLGNAGIKLWVLTGDKVETAINIGFSCNLLNNDMELIHLKVDEDESGEITDEAFFEM
AN8672.1  	TISLLARAGIKLWVLTGDKVETAINIGFSCNLLTNEMELIVFNIPGDQR---------HQ
          	:* :*..**************************.*:*:*: :::  *:            

MG02767.1 	LEAELDKYLKIFNLTGSDEDLAAARKIHEAPQATHAVVIDGFTLRWVLEDTLKQKFLLLC
NCU07443.1	AEEELDRGLAKFGMTGSDEELRQAKKDHEPPAPTHGLVVDGFTLRWVLSDTLKQKFLLLC
FG08595.1 	AEKLLDDNLQIFGITGSDHDLALAKKNHEPPAPTHGLVIDGFTLRWVLNDRLKQKFLLLC
AN8672.1  	ASRELDEHLRKFQLTGSDEELIEARQNHKPPEPTHAVVIDGETLKLMLSDEMKQRFLLLC
          	 .  **  *  * :****.:*  *:: *:.* .**.:*:** **: :*.* :**:*****

MG02767.1 	KQCKSVLCCRVSPAQKAAVCAMVKNGLDVMTLSIGDGANDVAMIQEADVGVGIAGVEGRQ
NCU07443.1	KQCKSVLCCRVSPAQKAAVVSMVKNGLDVMTLSIGDGANDVAMIQEADVGVGIAGEEGRQ
FG08595.1 	KQCKSVLCCRVSPAQKAAVVAMVKNGLDVMTLSIGDGANDVAMIQEADVGVGIAGVEGRQ
AN8672.1  	KQCKSVLCCRVSPAQKAAVVKLVKDGLNIMALSIGDGANDVAMIQAADVGVGIIGEEGRQ
          	*******************  :**:**::*:************** ******* * ****

MG02767.1 	AVMSSDYAIAQFSFLQRLVLVHGRWSYRRLAESISNFFYK~NLVWAMPLFLFQIYCDFDM
NCU07443.1	AVMSSDYAIGQFRFLTRLVLVHGRWSYRRLAETISNFFYK~NMVWTWAIFWFQIFCDFDI
FG08595.1 	AAMSSDYAIAQFRFLSRLVLVHGRWSYRRLAESISNFFYK~NMVWTFSIFWYEIYCDMDM
AN8672.1  	AAMSADYAIGQFRFLQRLILVHGRYSYRRLGETTANFFYK0NLVWTFALFWYSIYNDFDG
          	*.**:****.** ** **:*****:*****.*: :***** *:**: .:* :.*: *:* 

MG02767.1 	TYLFDYTYILMFNLLFTSVPVILMGVLDQDVSDTVSLAVPQLYRRGIERLEWTQTKFW2L
NCU07443.1	SYIFDYTYILMFNLFFTSIPVILMGVLDQDVSDTVSLAVPQLYRRGIERLEWTQTKFW2A
FG08595.1 	TYLFDYTYILMFNLFFTSIPVAIMGVLDQDVSDKVSLAVPQLYRRGIERLEWTQLKFW2L
AN8672.1  	SYLFDYTYIILVNLAFTSLPVILMGIFDQDVDDKVSLAVPQLYMRGIERLEWSQAKFW2L
          	:*:******::.** ***:** :**::****.*.********* ********:* ***  

MG02767.1 	YMLDGTYQGVMSFFIPYLVVVGSPFVTTNGLDVTDRVRFGAYIAHPAVVTINLYILINSY
NCU07443.1	YMADGIYQSVMSFFIPFIFCILTPAASGNGLDVQERTRLGCYIAHPAVLTINMYILMNTY
FG08595.1 	YMIDGIYQSIMVFFIPYLLFMPGTFLTGNGLGLEDRLRFGTYVAHPAVITINMYILINTY
AN8672.1  	HMADGFYQSVICFYMPYLLYEPANFVTENGLDVSDRNRMGILVASCAVIASNTYILMNSY
          	:* ** **.:: *::*::.       : ***.: :* *:*  :*  **:: * ***:*:*

MG02767.1 	QWDWLIVLVVVLSDLFVFFWTGVFTSNTYSQWFYEAAPQIYAQPSFWAVFIITPVMCMFP
NCU07443.1	RWDWVMLLVVFLSDIFIFFWTGIYTATSYSGQFYQAAPQVYAEFTFWMAFIITPTICLLP
FG08595.1 	RWDWLMVLIVVISDVFIFFWTGVYTSFTSSQYFYGTAAQVYGEATFWACFFLVPVICLFP
AN8672.1  	RWDWLTVLINAISCLLIFFWTGVYSSVQASAQFYKSAAQTYGTLTFWVVLLLTVTICLLP
          	:***: :*:  :* :::*****::::   *  ** :*.* *.  :**  :::. .:*::*

MG02767.1 	RFSIKALQKVYWPYDVDIIREQVQQGKFDRLQP---APVGQTLPGGRSTAGSSPSLAKGN
NCU07443.1	RLVTKCIQKQMFPYDVDIIRERISTGEYKAIEVPVVAPVEDEALKGTSSGSSESSSSRAK
FG08595.1 	RFAIKALQKVYWPYDVDIIREQERMGNFAYLDK--TDETNDPPTADTKSDKSKSSKSSRR
AN8672.1  	RFVVKSVQKVFFPLDVDIIREQITQGKFKYLDQYETFVPKAAGSANDSAASSDLGKPVDS
          	*:  *.:**  :* *******:   *::  ::             . .:  *. . .   

MG02767.1 	PG--------TFSGQDDE-RPIYPPS-----VATHTRTQTGSDGTNYTGHRMSMEQQQLE
NCU07443.1	SKSSKHSKHTHYASVDEDRRPIYPPS----VNTHHTRAQNGSDGTNYTRHSLDTNQIEDE
FG08595.1 	SK--KMPKHVAYGSVDEDLRPIYPPSTATRATTYNQHSQNGSDSTNYTAH-----RISLD
AN8672.1  	GT--------RHNPFSDD-QQIYAESVAPTVRTHNPRGQNGSNGTTYASSIDTTRHPQPQ
          	           .   .:: : **. * :.   : : : *.**:.*.*:       : . :

MG02767.1 	HQPLDQLTPMEHIPTRPSIDRARPSYDRVRASMDR--VRPSYEASNDFTSAARLSRVESS
NCU07443.1	-------PPMGGG--RPSIDRARPSYDRLRRSMDRERIRQSLEQSNDFTSAARLSRIEST
FG08595.1 	-------VPMQG---RPSIDRARPSYDRMRASMDR--VRPSFEASNDFTSAARLSRIESS
AN8672.1  	-----------------AVDYVRSSADRTRHSFDR--ARQSFEAVDGFASANTLTRVESS
          	                 ::* .*.* ** * *:**   * * *  :.*:**  *:*:**:

MG02767.1 	RSRGGPPPSS----HNLFAGRLRGLSIISAKSRHGPDSRGGQ-------------
NCU07443.1	HSAQNLPTPGAQRRFNLTTVRKRGLSVFSKQSEDEVPHPGVEDEDKRDERRDSQQ
FG08595.1 	QSQTGRFHP-----------RLRGLSLTKSANI----------------------
AN8672.1  	LTTG----------------AHRPQSPHSPLKSN---------------------
          	 :                    *  *  .  .
```
